# Genetic protection from type 1 diabetes resulting from accelerated insulin mRNA decay

**DOI:** 10.1016/j.cell.2025.02.018

**Published:** 2025-03-19

**Authors:** René van Tienhoven, Denis O’Meally, Tristan A. Scott, Kevin V. Morris, John C. Williams, John S. Kaddis, Arnaud Zaldumbide, Bart O. Roep

**Affiliations:** 1 Department of Diabetes Immunology, Arthur Riggs Diabetes and Metabolism Research Institute, Beckman Research Institute, City of Hope, Duarte, CA 91010, USA; 2 Department of Cell and Chemical Biology, Leiden University Medical Center, Leiden 2333ZA, the Netherlands; 3 Department of Diabetes and Cancer Discovery Science, Arthur Riggs Diabetes and Metabolism Research Institute, Beckman Research Institute, City of Hope, Duarte, CA 91010, USA; 4 Center for Gene Therapy, Beckman Research Institute, City of Hope, Duarte, CA 91010, USA; 5 Centre for Genomics and Personalised Health, School of Biomedical Sciences, Queensland University of Technology, Kelvin Grove, Brisbane, QLD 4059, Australia; 6 Department of Molecular Medicine, City of Hope National Medical Center, Duarte, CA 91010, USA; 7 Department of Internal Medicine, Leiden University Medical Center, Leiden 2333ZA, the Netherlands; 8 Lead contact

## Abstract

Insulin gene (*INS*) variation and beta-cell stress are associated with the risk of development of type 1 diabetes (T1D) and autoimmunity against insulin. The unfolded protein response alleviating endoplasmic reticulum (ER) stress involves activation of inositol-requiring enzyme 1α (IRE1α) that impedes translation by mRNA decay. We discover that the IRE1α digestion motif is present in insulin mRNA carrying SNP rs3842752 (G>A). This SNP in the 3′ untranslated region of *INS* associates with protection from T1D (*INS*^*P*^). ER stress in beta cells with *INS*^*P*^ led to accelerated insulin mRNA decay compared with the susceptible *INS* variant (*INS*^*S*^). Human islets with *INS*^*P*^ showed improved vitality and function and reversed diabetes more rapidly when transplanted into diabetic mice than islets carrying *INS*^*S*^ only. Surrogate beta cells with *INS*^*P*^ expressed less ER stress and INS-DRiP neoantigen. This explanation for genetic protection from T1D may act instead of or in concert with the previously proposed mechanism attributed to *INS* promoter polymorphism.

## In brief

Insulin gene variation predisposes individuals to type 1 diabetes (T1D). We report a new mechanism of genetic protection from T1D in which insulin mRNA with a protective SNP is broken down by IRE1α, thus alleviating ER stress, leading to better human islet function, fewer neoantigens and immunogenicity, and less autoimmunity, and ultimately reducing the risk for T1D.

## Graphical Abstract

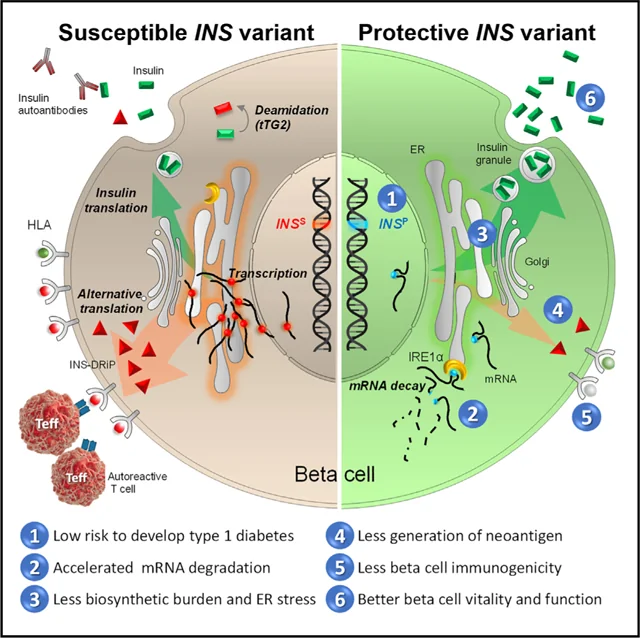

## INTRODUCTION

Type 1 diabetes (T1D) is a multifactorial autoimmune disease with a strong genetic predisposition leading to the destruction of insulin-producing pancreatic beta cells and loss of glycemic control.^1^ Besides human leukocyte antigen (HLA) genes, genetic risk for T1D is strongly associated with insulin gene (*INS*) varia-tion,^2^ including a variable number of tandem repeats (VNTRs) in the promoter region and single nucleotide polymorphisms (SNPs) in the 3′ UTR of *INS* that are in tight linkage disequilibrium (LD).^3^ These genetically linked variants (rs689, rs3842752, and rs3842753) have therefore been used interchangeably to tag *INS* variation, rather than to identify potentially causal protective polymorphisms. Previous models proposed that VNTR variation in the *INS* promoter region protects against T1D by increasing thymic expression while decreasing pancreatic expression of proinsulin, thereby enhancing central immune tolerance involving thymic deletion of autoreactive T cells.^4,5^ Indeed, pa-tients developing T1D despite carrying *INS*-associated genetic protection show decreased T cell autoimmunity and islet autoantibodies against insulin and proinsulin.^6,7^ Yet, this promoter variation was often defined by tagging SNPs instead (e.g., rs689 or rs3842752).^3,5–8^ The hypothesis of central tolerance is inconsistent with more recent observations that T1D patients carrying protective *INS* gene variation (represented by rs689) can display T cell autoimmunity to insulin, be it that this is characterized by immune regulatory IL-10 production.^6^ This implies peripheral tolerance rather than central tolerance.

*INS* gene variation also features prominently in the increasingly popular genetic risk scores used to predict or diagnose T1D, where rs689 was selected for GRS1 and rs3842753 for GRS2, without particular justification.^9,10^ *INS* variation tagged by rs689 also functions as a correlate of residual beta-cell mass and time in glycemic range in T1D patients.^7,11–13^ This is consistent with a lower risk of developing diabetic complications that we recently reported for patients with the protective *INS* variant on the basis of rs3842752.^14^ In the context of clinical trials aiming to prevent T1D, children with the susceptible *INS* variation (genotyped by rs689) developed insulin autoantibodies in response to oral insulin treatment.^15^ These children also displayed microbiome alterations linked to T1D development. Curiously, children carrying the protective *INS* variant rarely developed insulin antibodies after oral insulin treatment and showed reduced numbers of effector T cells and increased numbers of regulatory T cells to insulin in comparison with those homozygous for the susceptible *INS* variant, extending the potential relevance of *INS* variation to immune intervention and precision medicine. This observation is in line with peripheral immune regulation rather than central tolerance as an explanation of *INS*-related genetic protection, underscoring the need for alternative mechanisms to explain reduced risk.

The differences in *INS* expression in beta cells related to *INS* variation remain poorly understood, but a key role of beta cells in contributing to their own demise becomes increasingly evident. The insulitic lesions in the pancreas of T1D patients are characterized by upregulated HLA class I expression, production of chemokines, and endoplasmic reticulum (ER) stress.^1^ Single-cell transcriptomics suggested increased transcription of ER stress genes in beta cells of non-diabetic and type 2 diabetic donors carrying rs3842753 susceptible *INS* variants.^16^ ER stress in beta cells is believed to trigger islet autoimmunity.^1^ We recently described the generation of a stress-induced alternative *INS* product due to ribosomal infidelity that can act as an immunogenic neoantigen.^17^ This defective ribosomal *INS* product (INS-DRiP) results from alternative initiation of translation of insulin mRNA in the +2 reading frame, thereby skipping the canonical stop codon and resulting in translation of two SNPs (rs3842752 and rs3842753) in the *INS* 3′ UTR that strongly correlate with protection from T1D into polymorphic proteins. CD8 T cells isolated from T1D patients that are reactive with INS-DRiP selectively destroy stressed beta cells.^17^ Importantly, CD8 T cells have been isolated from pancreatic islet lesions of T1D organ donors that respond to preproinsulin (PPI) or INS-DRiP, indicating the importance of INS-DRiP reactivity in T1D pathology.^18^ Again, the rare cases developing T1D despite carrying a protective *INS* variant never showed T cell responses to INS-DRiP.^17^ SNP rs3842752 drew our attention following our discovery that this SNP is translated into INS-DRiP protein as a neoantigen, following beta-cell stress, while this SNP was often dismissed or ignored because of its strong LD with rs689, rs3842753, and the VNTR in the *INS* promoter and its location in the 3′ UTR of *INS*. Since central tolerance against a neoantigen expressed in stressed islets but not in the thymus is inconceivable, we tested the hypothesis that autoimmunity to INS-DRiP and other *INS* products in individuals with protective *INS* variants is controlled by peripheral immune tolerance, possibly acting in concert with central immune tolerance to proinsulin itself.

To alleviate ER stress and the accompanying INS-DRiP production, beta cells initiate the unfolded protein response (UPR) involving activation of inositol-requiring enzyme 1α (IRE1α). ER stress activates IRE1α by promoting dimerization and autophosphorylation, resulting in splicing of X-box binding protein 1 (XBP1) mRNA into XBP1s and increased ER chaperone expression, degradation of misfolded proteins, and reduced translational burden by regulated IRE1α-dependent decay (RIDD) of mRNA.^19,20^ This RIDD process requires a specific mRNA motif (CUGCAG) and hairpin structure to act as a substrate of IRE1α.^21,22^ We tested whether human insulin mRNA variants can be a substrate for IRE1α, leading to insulin mRNA decay, decreased ER stress and neoantigen formation, and improved beta-cell function and vitality.

## RESULTS

### “Protective” human insulin mRNA can be cleaved by IRE1α and decays more rapidly during ER stress

The *INS* 3′ UTR contains two proximate SNPs (rs3842752 and rs3842753) generating variation of insulin mRNA, the U-A variant associated with protection from T1D (designated *INS*^*P*^) and the “susceptible” C-C variant (*INS*^S^).^17^ Protection of *INS*^*P*^ is dominant in *INS*^*P/S*^ heterozygous individuals.^2,3^ The secondary structure of both mRNA variants was derived from *in silico* modeling.^23^ Protective insulin mRNA contains the CUGCAG motif and is able to form the hairpin structure required for RIDD, whereas susceptible insulin mRNA neither contains the IRE1α substrate motif nor forms the hairpin loop (Figure 1), implying that only *INS*^*P*^ mRNA fulfills both preferences to be recognized and cleaved by IRE1α.

We performed an *in vitro* IRE1α cleavage assay with human insulin mRNA derived from human islets isolated from pancreas donors. Total mRNA from homozygous susceptible (*INS*^*S/S*^), heterozygous protective (*INS*^*P/S*^), and homozygous protective islet donors (*INS*^*P/P*^) was incubated with and without IRE1α, followed by insulin-specific qRT-PCR. Insulin mRNA from *INS*^*P/P*^ or *INS*^*P/S*^ islets was cleaved faster and significantly more often compared with mRNA derived from *INS*^*S/S*^ islet donors (Figure 1). Our findings reveal that IRE1α can cleave human insulin mRNA and that cleavage of protective insulin mRNA is increased and faster than the susceptible mRNA variant.

Next, we determined the effect of *INS* variation on mRNA stability during ER stress using a dual luciferase reporter assay. The *INS* 3′ UTR was cloned downstream of *Renilla* luciferase (*R*luc) to generate two vectors, either carrying protective *R*luc-INS^P^ or susceptible *R*luc-INS^S^. Both vectors also coded for firefly luciferase to normalize their transfection efficiencies (Figures 2A and S1). These vectors were transfected into human embryonic kidney (HEK) 293T cells, and ER stress was induced with increasing amounts of thapsigargin, followed by comparison of *Renilla* and firefly luciferase activity. The decline in *Renilla* luciferase activity was significantly larger in cells transfected with *R*luc-INS^P^ compared with *R*luc-INS^S^ (Figure 2B). The difference between *R*luc-INS^P^ and *R*luc-INS^S^ breakdown was not observed in the presence of IRE1α inhibitor MKC3946 (Figure 2C). These results imply that the protective *INS* 3′ mRNA is less stable during ER stress and that this breakdown is mediated by IRE1α. RIDD of the protective insulin mRNA variant may thus reduce the translational burden during ER stress, which implies that ER stress can be resolved more easily in beta cells carrying the *INS*^*P*^ variant. Of note, the *INS* promoter region containing the VNTR was excluded from this study design, implying that the observed effect is solely mediated by the SNP in the *INS* 3′ UTR.

### Insulin mRNA levels correlate inversely with beta-cell stress only with *INS*^*P*^

The prevailing hypothesis of *INS*-related protection from T1D proposed that reduced risk stems from reduced transcriptional activity in the pancreas caused by the insulin promoter polymorphism (class III VNTR). We challenge this notion with our novel hypothesis that protection results from IRE1α-mediated decay of insulin mRNA of the protective *INS* variant during stress. We performed single-cell RNA (scRNA) transcriptomic studies on dispersed human pancreatic islets isolated from non-diabetic donors that we genotyped for *INS* SNP rs3842752 and matched for BMI and age, yielding eight heterozygous protective donors (*INS*^*P/S*^) and eight homozygous susceptible donors (*INS*^*S/S*^). Homozygous protected *INS*^*P/P*^ donors were unavailable. Clinical and laboratory characteristics of organ donors were similar between genotypes (Table S3). Cell types of the pancreatic islets were assigned as described previously^24^ (Figure S2). Nascent insulin RNA counts (unspliced pre-RNA) and mature spliced insulin mRNA were distinguished as previously described^25^ to test for differential *INS* promoter activity and mRNA content, respectively. Nascent insulin RNA counts did not differ between donors expressing the protective versus susceptible *INS* variant (Figure 3), implying no difference in transcriptional activity between *INS* variants in pancreatic beta cells. Mature insulin mRNA counts were also similar between *INS* genotypes in beta cells. Yet, when beta cell stress was taken into consideration, we found an inverse correlation between insulin mRNA levels and beta cell stress but only in donors expressing the protective *INS* variant: as stress increased, insulin mRNA decreased (Figure 3). By contrast, insulin mRNA levels were unaffected by beta-cell stress in donors only expressing the susceptible *INS* variant. Control genes unrelated to ER stress showed no difference between variants (e.g., protein kinase G [*PKG1*], *p* = 0.622; succinate dehydrogenase complex flavoprotein subunit A [*SDHA*], *p* = 0.311; and tubulin beta [*TUBB*], *p* = 0.102). These findings support our hypothesis that the protective *INS* SNP rs3842752 variant enables the breakdown of insulin mRNA during beta-cell stress.

The differential gene expression analysis revealed that beta cells carrying *INS*^*P*^ expressed lower levels of *TGM2*, which encodes tissue transglutaminase-2 (Figure 3). The potential clinical relevance of *TGM2* expression in beta cells was assessed in the context of T1D immunopathogenesis, where two beta-cell subsets were identified, of which the minor beta-2 subset was characterized by specific upregulation of apoptotic and adaptive immune system signaling pathways and was shown to be preferentially destroyed in pancreata of T1D patients.^26,27^ We used their publicly available single-cell multi-omics analysis to discover that the beta-2 subset had 1.7 times higher levels of *TGM2* (false discovery rate [FDR] 8.1 3 10^−36^) compared with the beta-1 subset. In turn, *TGM2* expression in beta-2 subset of T1D patients was significantly higher compared with non-diabetic individuals with (four times higher) or without islet autoantibodies (two times higher) (FDR 4.6 3 10^−18^ and 0.02, respectively). We propose that lower levelsof *TGM2* in beta cells carrying *INS*^*P*^ associate with reduced immunogenicity and beta cell survival in the context of T1D immunopathogenesis. LncRNA AC114971.1 was higher in beta cells with *INS*^*P*^ and has been implicated in type 2 diabetes in genome-wide association study (GWAS) studies.^28^

### Improved vitality and function of human islets carrying the protective *INS* variant

*Ex vivo* islet vitality and function were determined by measuring oxygen consumption rate (OCR) and glucose-induced insulin secretion. Human islets carrying one or two copies of *INS*^*P*^ showed a significantly higher glucose-stimulated change in OCR compared with islets homozygous for *INS*^*S*^ (Figures 4A and 4B), indicative of greater vitality and activity. Accordingly, is-lets with *INS*^*P*^ secreted more insulin under low and high glucose conditions compared with islets carrying *INS*^*S*^ only (Figure 4C), with similar stimulation indices. Thus, islets with *INS*^*P*^ showed better metabolic function and more insulin secretion, suggesting increased capability to respond to energetic demand (such as high glucose levels) and regulate blood glucose levels.

To evaluate *in vivo* islet function, human islets were transplanted into non-obese diabetic (NOD) severe combined immunodeficiency (SCID) mice made diabetic by streptozotocin treatment, and blood glucose levels were monitored for 4 weeks post-transplantation. Animals that reversed diabetes became normoglycemic twice faster, when they were transplanted with islets carrying *INS*^*P*^ compared with *INS*^*S*^ only (diabetes remission within 13 vs. 26 days, respectively; Figures 4D–4F). Diabetes reversal rates were similar between the protective and susceptible *INS* variant (14 out of 21 [67%] vs. 10 out of 16 [63%], respectively). These results point to improved *in vivo* function and accelerated diabetes reversal by islets carrying *INS*^*P*^. Of note, clinical and laboratory characteristics of organ donors were similar between the protective and susceptible group (Table S4).

Homozygosity for *INS*^*P*^ is rare (1%–4%). Fortunately, one pancreas donor proved homozygously protected. Although anecdotal, islets from this donor showed consistently higher rates of vitality and function. Insulin mRNA from the homozygous protective donor was cleaved even more and faster than that of heterozygous donors (Figure 1). Additionally, islets from the *INS*^*P/P*^ donor had the highest OCR fold change (1.57 pmol/min/islet equivalent [IEQ]; Figure 4B) and the highest insulin secretion in both low and high glucose conditions (mean of 10.8 and 15.2 mIU/mL/IEQ; Figure 4C) and reversed diabetes in mice *in vivo* in 4 or 8 days (Figure 4F). These results suggest a gene dosing effect of the protective insulin allele, in which homozygous protective islets function superior to heterozygous protective islets, which is consistent with increased RIDD of insulin mRNA.

### Lower neoantigen production and ER stress with *INS*^*P*^

To test the expression of neoantigen INS-DRiP in relation to *INS* variation, surrogate beta cells were created by transfecting HEK293T cells with INS-DRiP-GFP fusion constructs either carrying the protective or susceptible UTR, where the GFP coding sequence was positioned in frame with INS-DRIP. These constructs lead to stable PPI expression and variable GFP expression depending on stress-related translation initiation at the alternative AUG, as described before.^17^ INS-DRiP-GFP expression in surrogate beta cells with either *INS* variant during ER stress was tested by flow cytometry. The percentage of INS-DRiP-GFP+ cells and the mean fluorescence intensity (MFI) of INS-DRiP-GFP were significantly lower in *INS*^*P*^ surrogate beta cells compared with *INS*^*S*^ (Figure 5). By contrast, the percentage of C-peptide-positive cells was similar between *INS*^*P*^ and *INS*^*S*^ surrogate beta cells (Figure S3). When IRE1α was inhibited with MKC3946, no difference in INS-DRiP-GFP expression was observed between *INS*^*P*^ and *INS*^*S*^ (Figure 5A). Next, we determined the difference in INS-DRiP-GFP expression between *INS*^*P*^ and *INS*^*S*^ with and without IRE1α inhibition to show that this difference in INS-DRiP-GFP expression was significantly larger when IRE1α was not inhibited (Figure 5C). These results indicate that carrying *INS*^*P*^ leads to lower neoantigen expression compared with *INS*^*S*^ and that this process is mediated by IRE1α. Of note, expression of INS-DRiP-GFP went up when IRE1α was inhibited, possibly due to additional stress mediated by inhibition of the UPR.

To determine whether the protective *INS* variant associates with reduced biosynthetic burden, we measured mRNA levels of the canonical ER stress marker XBP1s in surrogate beta cells that were created by transducing K562 cells with *INS*^*P*^ or *INS*^*S*^ coding sequence. ER stress was induced by subsequent transduction with a shRNA-targeting BiP (shBiP), which led to 50%–60% knockdown of BiP gene expression (Figure 6A). BiP knockdown led to IRE1α activation as shown by increased levels of XBP1-spliced mRNA compared with the control short hairpin. XBP1-spliced levels were lower in surrogate beta cells that were transduced with *INS*^*P*^, compared with *INS*^*S*^ (Figure 6B). These results are consistent with lower levels of ER stress markers IRE1α and ATF6 in human beta cells associated with another protective *INS* SNP variant (rs3842753) that is in strong LD with rs3842752.^16^

## DISCUSSION

Protection from T1D inferred by *INS* variation has previously been claimed to be attributable to central tolerance, in which class III VNTR alleles are associated with increased thymic expression of insulin mRNA.^3,5,6,8^ We present a novel explanation for genetic protection from T1D inferred by polymorphisms in the *INS* 3′ UTR involving peripheral tolerance, which may act in concert with, or instead of, the previously proposed mechanism of central tolerance. Due to high LD between the VNTR in the promoter region and SNPs in the 3′ UTR of *INS*, it is conceivable that this newly identified mechanism of genetic protection explains the protection previously ascribed to the *INS* promoter,^29^ notwithstanding that polymorphisms in the promoter region and 3′ UTR of *INS* may act synergistically. Yet, our observation that *INS* variation did not affect nascent insulin RNA expression implies that the VNTR in the *INS* promoter region cannot explain lower levels of insulin mRNA in beta cells expressing the protective *INS* variant. Instead, it is conceivable that differences in *INS* mRNA content in stressed beta cells result from variation in the *INS* 3′ UTR, and that SNP rs3842752 is the causal variant explaining protection from the beta-cell perspective. Although we cannot rule out that thymic *INS* expression contributes to genetic protection from T1D and thus acts in concert with the protection that we attribute to SNP rs3842752. Our discovery of RIDD of human insulin mRNA provides an alternative explanation for the lower levels of the protective insulin mRNA variant found in the pancreas^5^ and the accompanying lower levels of ER stress markers in protected beta cells.^16^

RIDD of mRNA can reduce the translational burden in stressed cells. Insulin mRNA can make up to 38% of the beta-cell transcriptome or 100,000–200,000 copies per beta cell.^30,31^ Mouse insulin mRNA (mIns2) has been shown to be cleaved *in vitro* by IRE1α^32^ but lacks the genetic variation of human *INS*. We here demonstrate that IRE1α cleaves human insulin mRNA with the protective variant of *INS* SNP rs3842752 more often and more rapidly compared with the susceptible variant. Isolated human islets carrying the protective *INS* SNP rs3842752 displayed better cellular function, secreted more insulin, and reversed diabetes more rapidly in mice, compared with islets carrying the susceptible *INS* variant only. Using single-cell RNA transcriptomics of human islets, we demonstrate that stress in beta cells expressing the protective *INS* variant correlates inversely with insulin mRNA levels. This was not seen in beta cells from donors expressing only the susceptible *INS* variant. The reduced insulin mRNA content in stressed beta cells with *INS*^*P*^ was echoed by reduced expression levels of the stress-associated neoantigen INS-DRiP. Curiously, these beta cells expressed much lower levels of *TGM2*, which codes for tissue transglutaminase 2. This enzyme is known to post-translationally modify islet autoantigens by deamidation to create neoantigens with strong binding affinity to high-risk molecules of both HLA class I and II.^33,34^ Indeed, inflammatory stress-activated tissue transglutaminase-2 in human beta cells, creating neoantigens that are presented by HLA on the beta-cell surface. Strikingly, deamidated insulin B-chain stimulated proinflammatory autoreactive T cells of children suffering from T1D. The association between *TGM2* and T1D immunopathogenesis is supported by multiomics analysis in which we found higher TGM2 expression in a beta-cell subset (beta-2) that was shown to be preferentially destroyed in pancreata of T1D patients.^26,27^ Although transcriptomics cannot distinguish active from inactive tissue transglutaminase-2, our data support the notion that *INS*^*P*^ associates with less neoantigen formation, reduced immunogenicity, and superior vitality and function of beta cells.

Improved insulin secretion and more rapid loss of insulin mRNA may result from better beta-cell fitness attributed to alleviation of ER stress by IRE1α and imply that insulin mRNA may not be the limiting factor for insulin secretion. Furthermore, surrogate beta cells expressing the protective *INS* variant showed less ER stress and produced less neoantigen INS-DRiP during ER stress compared with surrogate beta cells with the susceptible *INS* variant. Since these surrogate beta cells contained the coding sequence of insulin with either SNP variant but lacked the VNTR variation in the *INS* promoter, this excludes the possible contribution by *INS* promoter polymorphisms and points to a causal, functional role of the SNPs in the *INS* 3′ UTR. The SNP in the 3′ UTR of *INS* is also present in an alternatively spliced insulin mRNA variant that we discovered recently.^35^ Therefore, RIDD of this alternatively spliced insulin mRNA would also affect the expression of this splice variant as neoantigen in beta cells and a subset of delta cells during ER stress in an SNP-dependent manner, and thereby further reducing islet immunogenicity and potentially affecting islet autoimmunity and immunopathology. We speculate that lower expression of the enzyme tissue transglutaminase-2, which can post-translationally modify insulin by deamidation into additional neoantigens, further adds to a reduced immunogenic face of beta cells carrying *INS*^P^. We propose that the reduced autoimmunity by CD4 T cells, CD8 T cells, and autoantibodies against natural and abnormal protein products of *INS* (proinsulin, insulin, and INS-DRiP) observed in T1D patients carrying the protective *INS* variant^6,7,17^ is a consequence of a combination of reduced ER stress, superior beta-cell vitality, and immune protection through reduced expression of autoantigens and neoantigen, mediated by accelerated decay of insulin mRNA by IRE1α. Indeed, inhibition of IRE1α with MKC3946 mitigated the effect of the *INS* variation on insulin mRNA breakdown and INS-DRiP expression. IRE1α inhibition increased expression of stress protein INS-DRiP, likely due to higher insulin mRNA levels and ER stress because the UPR was affected. These results indicate that IRE1α mediates insulin mRNA breakdown.

While the *INS* variation lies in the UTR of the canonical reading frame, stress-induced ribosomal infidelity leads to translation in the +2 reading frame coding for INS-DRiP.^17^ We previously reported that the N terminus of either INS-DRiP protein variant is naturally processed by both beta cells and antigen-presenting cells (APCs), leading to antigen presentation by HLA class I and class II, respectively. CD8 T cells of T1D patients recognizing the invariant N terminus of INS-DRiP_1–9_ that is identical between *INS*^*S*^ and *INS*^*P*^ destroyed human beta cells in a stress and inflammation-dependent fashion.^17^ Proliferative T cells (predominantly CD4) responding to APCs pulsed with recombinant INS-DRiP from T1D patients carrying the *INS*^*S*^ variant cross-reacted with both the susceptible and protective INS-DRiP variant, confirming that the CD4 T cell epitope is unlikely covering the INS-DRiP variant region (INS-DRiP_21–25_).^17^

A clinical benefit of carrying *INS*^*P*^ to the few patients developing T1D despite the associated protection has already been reported earlier in terms of lower incidence of insulin autoantibodies and, more importantly, higher C-peptide levels and lower hemoglobin (Hb) A1c.^7^ This could be explained by our newly proposed mechanisms of increased islet vitality and function and peripheral immune regulation. We recently showed that T1D patients carrying a copy of the protective *INS* variant had a reduced risk of developing diabetic complications.^14^ We speculate that the proposed mechanism of protective action associated with *INS* variation bears relevance to T1D heterogeneity, in particular, the recently proposed T1D “endotype 1” (T1DE1) that is characterized by early onset, extensive beta-cell destruction, aggressive insulitis, aberrant proinsulin processing, and high insulin autoantibody levels,^36,37^ compared with endotype 2 (T1DE2) or the general T1D population. A key characteristic of T1DE1 is the abnormal intracellular co-localization of proinsulin and insulin in beta cells in insulitic lesions, absent in T1DE2. This particular pathophysiological feature is highly reminiscent of biosynthetic burden, induction of ER stress, subsequent UPRs, and activation of IRE1α, which are key components in our proposed mechanism of genetic protection: in carriers of the protective *INS* variant, our proposed mechanism could alleviate ER stress through accelerated mRNA decay. In other words, cases carrying *INS*^*P*^ should be particularly unlikely to develop T1DE1, so the frequency of the protective *INS* variant should be even further reduced in T1DE1 cases or in patients who develop T1D under the age of 7 years, compared with its already reduced frequency among T1D patients or cases with T1DE2. This proposition is corroborated by the rarity of insulin autoantibodies in T1D cases carrying *INS*^*P*^, whereas these autoantibodies are increased in T1DE1.

Genotyping the *INS* 3′ UTR can help predict the function and vitality of human islets isolated for transplantation and serve as a prognostic biomarker for superior glycemic control and reduced risk for diabetic complications in T1D patients. Gene editing of the SNP could endorse autologous induced pluripotent stem cells (iPSCs) derived from T1D patients lacking this genetic protection with the benefits of *INS*^*P*^ by generating beta cells or pseudo islets harboring T1D-protective genetic traits of reduced stress and auto-immunogenicity and increased vitality and function.

This novel mechanism by which genetic risk polymorphism of *INS* contributes to islet autoimmunity puts beta cells at the heart of the immunopathology leading to T1D and adds to the accumulating evidence that beta cells are key contributors to their own demise. We propose that genetic risk predisposition modulates both beta-cell distress and associated immunogenicity, as well as the chance that this provokes the immune system to respond, leading to islet autoimmunity and T1D. This could explain why immune intervention therapy alone at best only delays progression of T1D.^1,38,39^ A complete arrest in disease progression and potential cure would then require beta-cell therapy, conceivably in combination with immunotherapy, to achieve durably restored beta-cell protection, vitality, and function. In the case of beta-cell replacement using stem-cell-derived beta cells, it seems attractive to generate stem cells genetically engineered by CRISPR-Cas editing to become homozygous for *INS*^*P*^, thus reducing immunogenicity and ER stress and increasing the stamina, functionality, and immune protection of the implanted beta cells.

### Limitations of the study

Even though we observed significant differences in mRNA decay and islet function and vitality between human islet donors carrying the protective *INS* variant versus the susceptible *INS* variant, the relative scarcity of pancreas donors and the related small sample size is a limitation of human islet studies. Homozygosity for the protective *INS* variant is rare (1%–4%), which adds to the challenges in our study. Our data imply that a single copy of *INS*^*P*^ suffices to alleviate stress, and it is conceivable that this inverse correlation between beta-cell stress and protective insulin mRNA is even stronger in homozygous *INS*^*P/P*^ individuals because all insulin transcripts would possess the digestion motif for IRE1α. Indeed, this notion is supported by the single islet donor who carried double protection out of the 34 cases studied. While our results warrant substantiation in larger cohorts of human pancreas donors, our claims are supported by results from independent studies pointing in the same direction, indicating that the protective *INS* variant associates with peripheral immune tolerance of proinsulin, lower levels of autoimmunity and beta-cell stress, and better beta-cell function and glucose regulation in T1D patients.

## RESOURCE AVAILABILITY

### Lead contact

Further information and requests for resources should be directed to and will be fulfilled by the lead contact, Bart O. Roep (boroep@lumc.nl).

### Materials availability

This study did not generate new, unique reagents.

### Data and code availability

The datasets generated and analyzed in this report are available from the lead contact upon request. Single-cell RNA sequencing data analyzed in this study are available without restriction at PANC-DB https://hpap.pmacs.upenn.edu/, the data portal of the Human Pancreas Analysis Program (HPAP; RRID: SCR_016202). Detailed clinical and other relevant organ donor-related data for scRNA-seq samples are also made available at PANC-DB, and clinical and laboratory characteristics and demographic data are reported in Table S3. Code for processing and analysis of HPAP scRNA-seq data is available via https://github.com/KaddisLab/2025_van_tienhoven_et_al_cell.

## STAR★METHODS

### EXPERIMENTAL MODEL AND STUDY PARTICIPANT DETAILS

#### Human islets

Human islets were acquired from Integrated Islet Distribution Program (IIDP) and cultured in Connaught Medical Research Laboratories (CMRL)-1066 media supplemented with 0.5% human serum albumin (Baxter Healthcare Corporation) and 0.1 μg/ml insulin-like growth factor-1 (Cell Sciences). Informed consent was obtained from the donor’s next of kin for the use of human tissue. All procedures were conducted in accordance with the City of Hope Institutional Review Board and the Declaration of Helsinki. Clinical and laboratory characteristics and demographic data is reported in Tables S3 and S4.

#### Cell lines

HEK293T (CRL-3216) and K562 cells (CCL-243) were obtained from ATCC and cultured in DMEM + 10% FBS + 1 μg/ml puromycin or IMDM + 10% FBS + 1 μg/ml puromycin, respectively. All islets and cells were cultured at 37°C in a humidified incubator with 5% CO_2_.

#### Mice

Male diabetic NOD SCID mice (15–25 weeks old) were treated with intraperitoneal injection of 50 mg/kg streptozotocin (Sigma-Aldrich) for three consecutive days and used to asses islet function. Animal studies were approved by the City of Hope Beckman Research Institute Institutional Animal Care and Use Committee.

### METHOD DETAILS

#### Human islets

Human islets were isolated as described previously.^40^ Donors were genotyped for rs3842752 and rs3842753 by Sanger sequencing using *INS*-specific primers (Fw: 5’-GGAGGGGCTCACAACAGTG and Rv: 5’-GTGTCTCCCTGACTGTGTCC). Islet preparations were used for DNA/RNA isolation using allprep DNA/RNA mini kit (Qiagen) and *ex vivo* and *in vivo* function analysis.

#### *In silico* RNA folding

Human insulin mRNA sequence variants with protective or susceptible 3’UTR were exported from the ensembl genome browser^41^ and folded *in silico* using mFOLD.^23^ Segments of the RNA were visualized using Forna.^42^

#### Cloning and dual luciferase reporter assay

The *INS* 3’ UTR (315–406 from insulin translation start) was inserted into pSI-Check 2.2 vector using NEBuilder HiFi DNA assembly cloning kit (New England Biolabs) after digestion with XhoI (New England Biolabs). Competent cells were transformed with the pSI-Check 2.2-INS 3’ UTR vector using NEBuilder HiFi DNA assembly chemical transformation protocol (New England Biolabs). Next, vectors were isolated using zymopure II plasmid midiprep kit (Zymo Reseach), and sequenced to validate the insert and absence of mutations. HEK293T cells were transfected with either vector using Lipofectamine 3000 (Invitrogen). After thapsigargin treatment (Ab120286, Abcam) and/or MKC3946 treatment (10 μM, Cayman Chemical), the dual-luciferase reporter assay was performed ac-cording to manufacturer instructions (Promega) and luciferase signal was detected on a Glomax Microplate Reader (Promega). Stability of the protective and susceptible *INS* UTR was determined by comparing *Renilla* luciferase activity with firefly luciferase activity.

#### *In vitro* cleavage assay and qRT-PCR

Whole islet mRNA (500 ng) was incubated with 0.5 mg recombinant IRE1α (custom made) in cleavage buffer (20 mM HEPES, 50 mM NaCl, 1 mM DDT, 1 mM ATP) at 37°C for 1h, followed by heat inactivation (5 min at 95^o^C). cDNA was made using SuperScript IV VILO (Invitrogen) and Taqman qPCR was done using TaqMan Fast Advanced (Applied Biosystems) and custom insulin specific primers and probe [Fw: 5’-CTGGAGAACTACTGCAACTAGAC; Rv: 5’-TGCTGGTTCAAGGGCTTTAT; and probe: 5’-CTCCTGCACCGAGAGAGATGGA (Applied Biosystems)].

#### Surrogate beta-cells

The pLV-CMV-INS-DRIP-GFP vectors (protective or susceptible) were digested using MfeI and the linear vector was purified using the DNA Clean & Concentrator-5 according to the manufacturer instructions (Zymo Research). HEK293T cells were seeded at 50,000 cells per well in a 48-well plate and 24h later, 1 μg of linear DNA was transfected into the cells using lipofectamine 3000 (Thermo Fisher Scientific). At 48h post-transfection the media was supplemented with 1 μg/ml puromycin (Gibco) to select for resistant clones.

#### Flow cytometry

Prior to flow cytometry, surrogate beta-cells were incubated with increasing concentration thapsigargin (Ab120286, Abcam) for 6h. IRE1α was inhibited by pretreatment with 50 μM MKC-3946 (19152, Cayman chemical). INS-DRiP-GFP expression was determined without fixation/permeabilization. Intracellular staining was performed to visualize C-peptide. Briefly, cells were fixed (00–5223-56, Invitrogen), permeabilized (00–8333-56, Invitrogen) and stained with rat-anti-C-peptide antibody (2 μg/ml, GN-ID4, DSHB, AB_2255626, kindly provided by O. Madsen) and visualized with donkey-anti-rat-AF647 (1 μg/ml, 715–605-151, AB_2340863, Jackson Immunoresearch). Cells were analyzed with the Attune Flow Cytometer (acoustic focusing cytometer, Invitrogen). The gating strategy is shown in Figure S4.

#### ER stress marker expression

To determine the effect of *INS*^*P*^ or *INS*^*S*^ on ER stress levels, surrogate beta-cells were created by lentiviral transduction of K562 cells with the *INS*^*P*^ or *INS*^*S*^ coding sequence. Subsequently surrogate beta-cells were transduced with a short hairpin RNA against BiP (shBiP, BiP knockdown) to induce stress or with a non-target short hairpin RNA control. The vectors were produced as described previously.^43^ Viral supernatants (MOI 1) were added to fresh medium supplemented with 8 μg/ml polybrene (Sigma-Aldrich) and the cells were incubated overnight. Medium was replaced the next morning. After two days, puromycin (2 μg/ml) selection was started for two days.

RNA was isolated from transduced K562 cells using NucleoSpin RNA kit (Bioke). 500 ng of RNA was used for reverse transcription using Oligo dT primers. Transcript expression was tested using iQ SYBR Green Supermix (BioRad) and the following primers; BiP forward GACGCTGGAACTATTGCTGG, BiP reverse CTCCCTCTTATCCAGGCCAT; XBP1us forward GGAGTTAAGACAGCG, XBP1us reverse CTGCAGAGGTGCACG; XBP1s forward CTGAGTCCGCAGCAG, XBP1s reverse GAGATGTTCTGGAGGGGTGA; GAPDH forward ACAGTCAGCCGCATCTTCTT, GAPDH reverse AATGAAGGGGTCATTGATGG

#### *Ex vivo* islet function

Islet oxygen consumption rate (OCR) was measured using a Seahorse XFe analyzer (Seahorse Bioscience). Briefly, islets were washed with modified Seahorse XFe assay media containing 3 mM glucose and 1% FBS, and equilibrated in same assay media for 3h at 37°C. Next, 70–100 IEQ islets were handpicked and plated into Seahorse XFe islet capture plates (Seahorse Bioscience). Islet OCR was measured at basal level (3 mM glucose), upon glucose stimulation (20 mM glucose), and on mitochondrial respiration inhibition (Oligomycin 5 nM). OCR fold increase was calculated by dividing OCR upon glucose stimulation by OCR at basal level. Minimum of 4 islet samples were measured simultaneously in each experiment.

For glucose-stimulated insulin secretion (GSIS) assay, 140 IEQ islets in triplicate were incubated with Krebs Ringer Buffer (KRB) containing 2.8 mM glucose for 1h followed by 28 mM glucose for 1h. Samples were collected at the end of each incubation, and insulin concentration was measured using the ELISA kit for human insulin (Mercodia). The stimulation index (SI) was calculated by dividing the total insulin released during the incubation with 28 mM glucose by the total insulin released in 2.8 mM glucose for the same period.

#### *In vivo* islet function

Transplantation of isolated human islets into male diabetic NOD SCID mice has been implemented by transplantation centers to assess islet function to reverse diabetes induced by intraperitoneal injection of 50 mg/kg streptozotocin (Sigma-Aldrich) for three consecutive days. 2–4 NOD SCID mice with hyperglycemia (>350 mg/dL) for at least two consecutive days were transplanted with identical numbers (1200 IEQ) of single donor human islets under the left kidney capsule. Blood glucose level was determined 2–3 times per week for 28 days using a glucometer (LifeScan Inc.). Transplant recipients that had two consecutive blood glucose measures <200 mg/dl were considered to have reversed diabetes and the first date of blood glucose measure <200 mg/dl was considered date of diabetes reversal. The reversal rate of diabetes was calculated based on the ratio of number of mice that reversed diabetes to that of total number of mice transplanted in each group.

#### Single-cell transcriptomics

Fastq files generated from the single cell RNA sequencing of hand-picked human pancreatic islets were downloaded from the data portal (i.e. PancDB) of the Human Pancreas Analysis Program (HPAP), as described elsewhere.^44,45^ For preprocessing we considered libraries from all donors, and then selected non-diabetic donors that passed quality control. Reads from libraries prepared using the 10x Genomics Chromium Single Cell Gene Expression 3’ v2, v3, or v3.1 chemistry kits were aligned with CellRanger v7.1.0 against the human reference genome GRCh38 (refdata-gex-GRCh38–2020-A) and intronic reads were counted.

Comprehensive library quality assessment was performed using the nf-core/scrnaseq pipeline.^46,47^ Samples that failed quality metrics including a low fraction of bases with Q-score ≥ 30 (HPAP-021, HPAP-038) or a low fraction of reads in cells (HPAP-023, HPAP-027, HPAP-093) were removed as reported previously.^24,48^ Cellbender (v0.3.2) was used to remove background noise arising due to ambient RNA and other technical factors.^49^ Doublets were identified using the default parameters of scDblFinder (v1.18.0)^50^ and discarded. To determine which cells to retain, we employed a data-driven approach with ddQC^51^ implemented in R (v4.4.1). For each library, cells were clustered using Seurat (v5.1.0), and quality metrics (UMI count, gene count, mitochondrial and ribosomal percentages) were evaluated. Cluster-specific thresholds were calculated using median absolute deviation (MAD). Cells were retained if they passed the ddQC metric thresholds (minimum 500 UMIs, 200 genes, > 5% ribosomal reads and fell within a 3.5 MADs for each metric). Given the focus on beta-cell stress, a relatively high threshold of 30% mitochondrial reads was used across all cells, which is comparable to published thresholds.^48^ Cell types were assigned using the default parameters of SingleR (v2.6.0)^52^ to transfer pruned labels from the Seurat object^53^ published by Elgamel et al.^24^ PCA was performed on the scaled expression matrix using the first 50 principal components. Harmony (v0.2.0)^54^ was used for batch correction with library chemistry and tissue source as covariates.^24^ UMAP dimensionality reduction and neighbor calculations were performed using the Harmony reduction. Clustering was performed using the Louvain algorithm through the igraph method at a resolution of 0.3. The tidyomics framework^55^ was used for data analysis, plotting and hypothesis testing.

Donors were genotyped using Phase 3 SNPs with allele frequencies > 5% from the 1000 Genomes Project [G].^56^ To derive SNP calls from scRNA-seq data we used cellsnp-lite (v1.2.3) in “Mode 1a” with BAMs from Cellranger and set the minimum minor allele frequency (MAF) threshold to 0.05 and a minimum read count threshold of 1000 for rs3842752. Donors were assigned homozygous reference (MAF < 0.3), heterozygous (0.3 ≤ MAF ≥ 0.7), or homozygous alternate (MAF R 0.7); representing the susceptible *INS* allele and protective *INS* allele, respectively. Cellranger BAMs were also used with STARsolo (v2.7.11b)^57^ to derive nascent and mature *INS* counts from intronic reads and exon-only reads respectively, using the default RNA velocity analysis with technology-specific white-lists (v2 or v3), that was previously described.^25^ A stress score was designed and customized to beta-cells on basis of previously identified stress correlates (ATF3, DDIT3 and TXNIP) in combination with a beta-cell specific correlate of inflammatory stress resulting in expression of an immunoproteasome unit specifically involved in processing of INS-DRiP (PSMB10^27^); the beta-cell stress score was calculated as the sum of counts of ATF3, DDIT3, PSMB10 and TXNIP.

To address compositional dominance of *INS* transcripts in beta-cells, *INS* expression was normalized to a set of constitutively ex-pressed genes whose expression levels are independent of *INS*. Abundance was calculated as the ratio of *INS* counts to the sum of counts of 11 housekeeping genes (actin beta [ACTB], glyceraldehyde-3-phosphate dehydrogenase [*GAPDH*], phosphoglycerate kinase 1 [*PGK1*], peptidylprolyl isomerase A [*PPIA*], ribosomal protein lateral stalk subunit P0 [*RPLP0*], succinate dehydrogenase complex flavoprotein subunit A [*SDHA*], transferrin receptor [*TFRC*], glucuronidase beta [*GUSB*], hydroxymethylbilane synthase [*HMBS*], hypoxanthine phosphoribosyltransferase 1 [*HPRT1*], and TATA-box binding protein [*TBP*]), scaled to 10000 and log-transformed; we calculated this for counts from Cellranger, and for nascent and mature counts from STAR-solo.

#### Donor Selection for single-cell transcriptomics

All scRNA-seq data available from individuals without diabetes and were considered for further analyses. Clinical parameters from organ donors with the protective vs. susceptible *INS* variant of rs3842752 were compared (data not shown). Since BMI was found to be statistically significantly different between the two groups, organ donors with the protective *INS* variant were BMI-matched to individuals carrying the susceptible variant. Matches were first made if BMI differed by only 1 unit, then by 2 units if no one was found (n=7 pairs; 14 individuals). If unsuccessful at a 2-unit BMI match, then a susceptible individual with the closet BMI and within 2 years of age of the protective carrier was selected (n=1 pair; 2 individuals).

### QUANTIFICATION AND STATISTICAL ANALYSIS

For all organ donor characteristics, percent was reported for categorical variables, mean ± SD for normally distributed continuous variables, and median (minimum, maximum) for continuous variables with a skewed distribution. Skewness was defined at values >2 and excess kurtosis >4, as previously described.^58^ For continuous variables, p-values were generated using a Satterthwaite-corrected t test if parametric or a Kruskal-Wallis test if nonparametric. For categorical variables, a Pearson chi-square test was used if values in all cells exceeded 5 or a Fisher exact test if one or more cells were <5; P values were not calculated if values from two or more cells were equal to 0. Analysis of Covariance (ANCOVA) was used to compare the slope of the regression lines using scRNA-seq generated expression data from those with the protective vs susceptible *INS* variant and the aov function in R version 4.3.1. All other analysis was performed using SAS Software (SAS Institute, Cary, NC) version 9.4.

All other statistical calculations were carried out using Graphpad Prism 9 (Graphpad software, San Diego, CA, USA). Statistical tests and presentation of data are indicated in the figure legends. P values of <0.05 were considered statistically significant.

## Supplementary Material

Document S1. Tables S1–S4

1

SUPPLEMENTAL INFORMATION

Supplemental information can be found online at https://doi.org/10.1016/j.cell.2025.02.018.

## Figures and Tables

**Figure 1.**
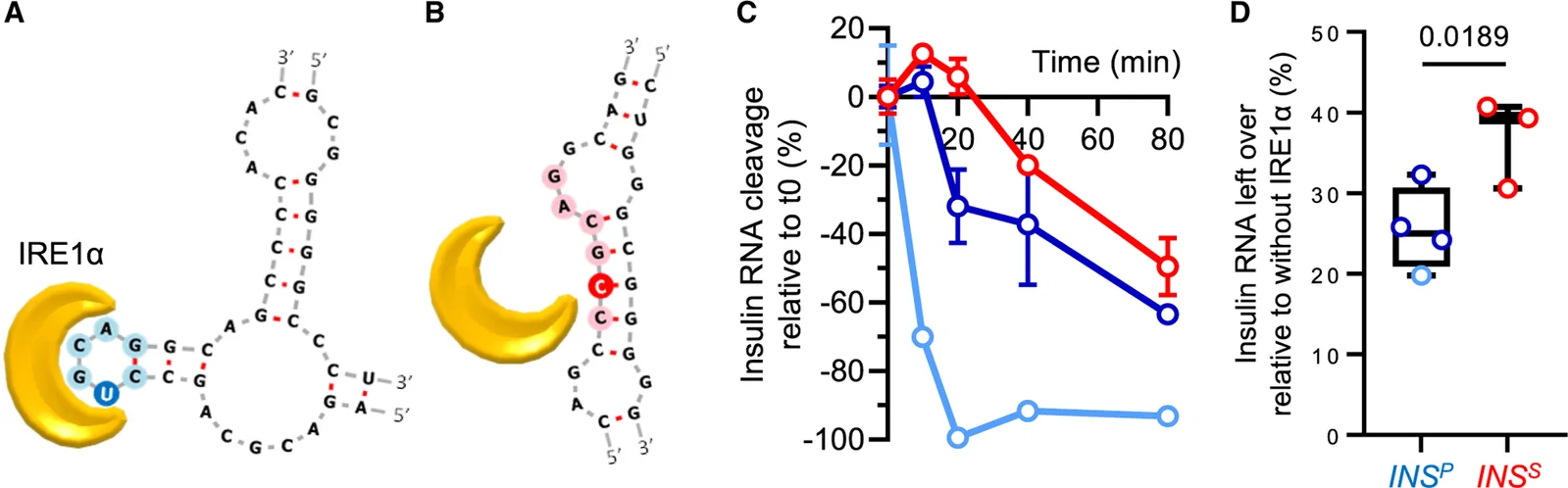
Protective human insulin mRNA contains an IRE1α motif and can be cleaved by IRE1α (A and B) The secondary structure of a segment of the *INS* 3′ UTR is shown for the protective (A) and susceptible (B) insulin mRNA variant. Colored bases form a hairpin loop in the protective variant (a, light blue) but not in the susceptible variant (b, light red). SNP rs3842752 is indicated by dark blue and dark red color. IRE1α is indicated by the yellow object. (C) Change in insulin mRNA levels after *in vitro* cleavage with recombinant IRE1α for one human pancreas donor with *INS*^*S/S*^ (red), *INS*^*P/S*^ (dark blue), or *INS*^*P/P*^ (light blue) over time, relative to time point 0. Delta Ct values were calculated by subtracting the mean Ct values of time point 0 from the other time points. Relative expression levels were calculated by 2^-dCt^, and the percentage insulin mRNA change was calculated by -(1–2^-dCt^) 3 100. All data points are displayed with error bars of their technical PCR replicates. (D) Percentage of insulin mRNA left over after 1 h *in vitro* cleavage with recombinant IRE1α for donors with *INS*^*S/S*^ (red, three donors), *INS*^*P/S*^ (blue, three donors), or *INS*^*P/P*^ (light blue, one donor). For statistical analysis, data from the single and rare homozygous *INS*^*P/P*^ islet donor were combined with those from heterozygous donors. Delta Ct values were calculated by subtracting the mean Ct values of RNA samples treated without IRE1α from samples treated with IRE1α. Percent relative expression was calculated by 2^−dCt^ × 100. All data points are shown, box and whiskers show min to max, and center line shows median. Significance was tested with one-tailed t test. See also Tables S1 and S2.

**Figure 2.**
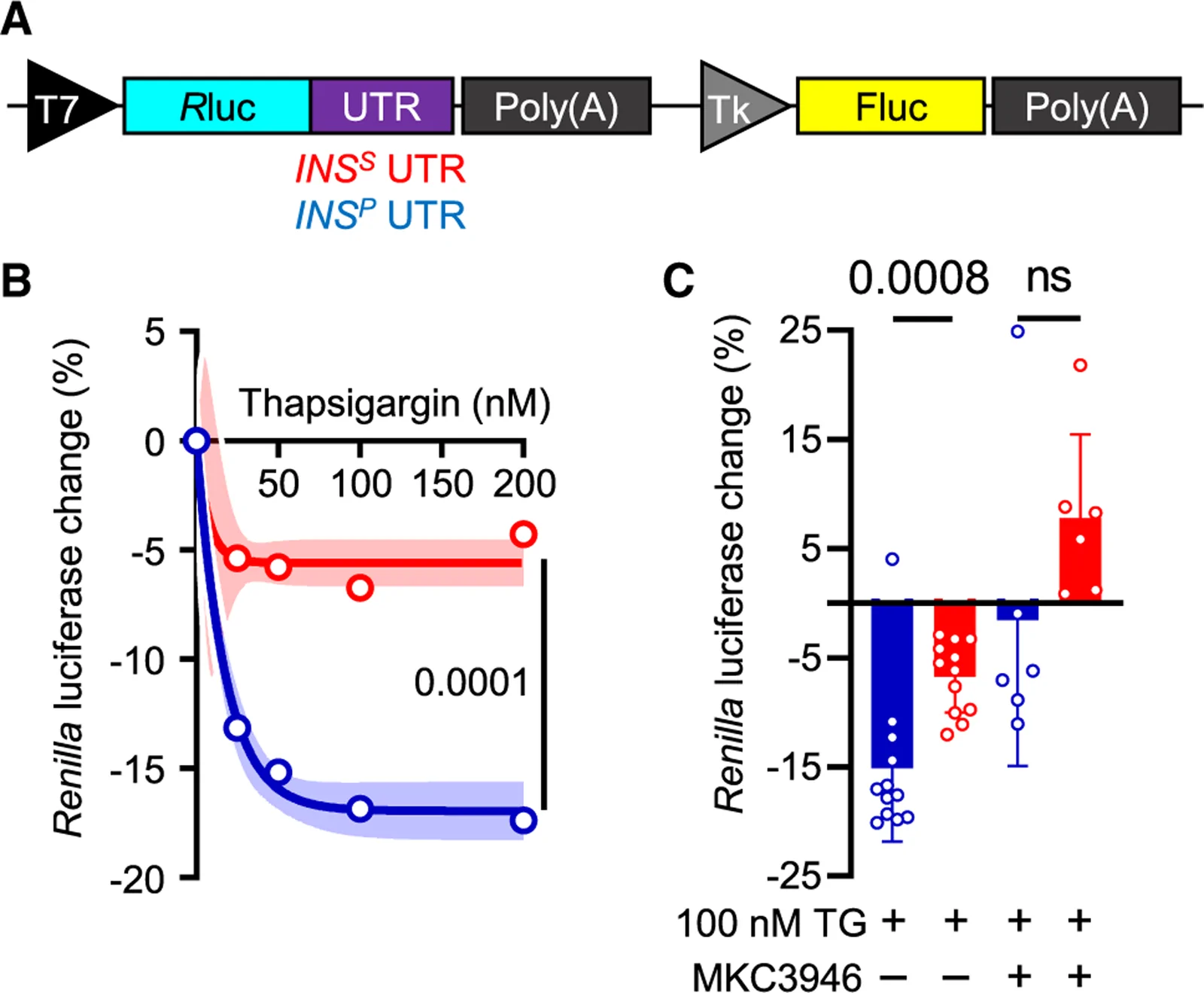
IRE1α mediates degradation of the insulin 3′ UTR during ER stress (A) Schematic overview of the dual luciferase vectors with the protective and susceptible *INS* 3′UTR inserted behind *Renilla* luciferase. (B) Change in *Renilla* luciferase activity with the protective (blue) or susceptible (red) *INS* 3′ UTR after thapsigargin-induced ER stress, normalized to firefly luciferase activity. Data of 3 independent experiments are shown as mean with 95% coincidence interval, and lines were fitted using nonlinear regression (one-phase decay). Significance was tested using a mixed model. (C) Change in *Renilla* luciferase activity after treatment with 100 nM thapsigargin with or without IRE1α inhibitor MKC3946 (10 mM). Data of 3 independent experiments are shown as mean ± SD. Significance was tested with two-tailed t test. See also Figure S1.

**Figure 3.**
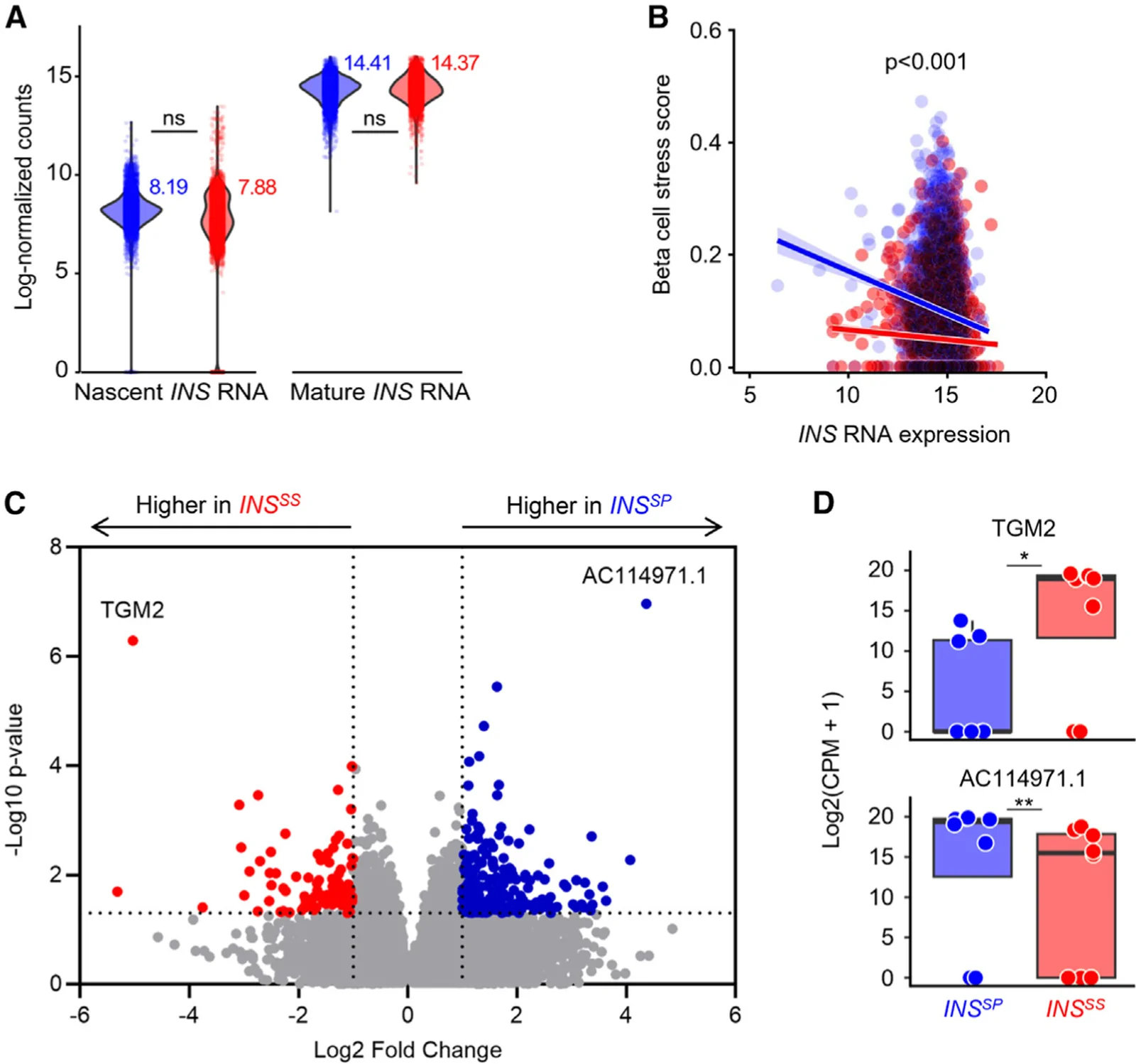
*INS* expression and differential gene expression in human beta cells with the protective or susceptible *INS* variants (A) Nascent and mature *INS* expression in beta cells with *INS*^*P/S*^ (blue) or *INS*^*S/S*^ (red). Violin plots show median expression values. Statistical analysis using linear mixed-effects models (with donor as a random effect) showed no significant differences (ns) between variants for either nascent or mature *INS* expression. (B) Correlation between beta-cell stress score and *INS* RNA expression. Scatter plot shows individual cells (*INS*^*S/S*^, red and *INS*^*P/S*^, blue) with fitted linear regression line and 95% confidence interval bands. *INS* expression levels inversely associate with beta-cell stress markers in beta cells with *INS*^*P/S*^ only. Correlations for beta cells with *INS*^*P/S*^ versus *INS*^*S/S*^ were significantly different from each other (*p* < 0.001). (C) Volcano plot of differentially expressed genes between *INS*^*P/S*^ or *INS*^*S/S*^, showing genes with higher expression in *INS*^*S/S*^ (red) or *INS*^*P/S*^ (blue). (D) Expression levels of top differentially expressed genes *TGM2* and AC114971.1 across donors. Box plots show median, quartile, and range of log_2_-normalized expression values for *INS*^*S/S*^ (red) or *INS*^*P/S*^ (blue) donors. Asterisks indicate statistical significance by Wilcoxon rank-sum test after multiple comparison correction. See also Table S3 and Figure S2.

**Figure 4.**
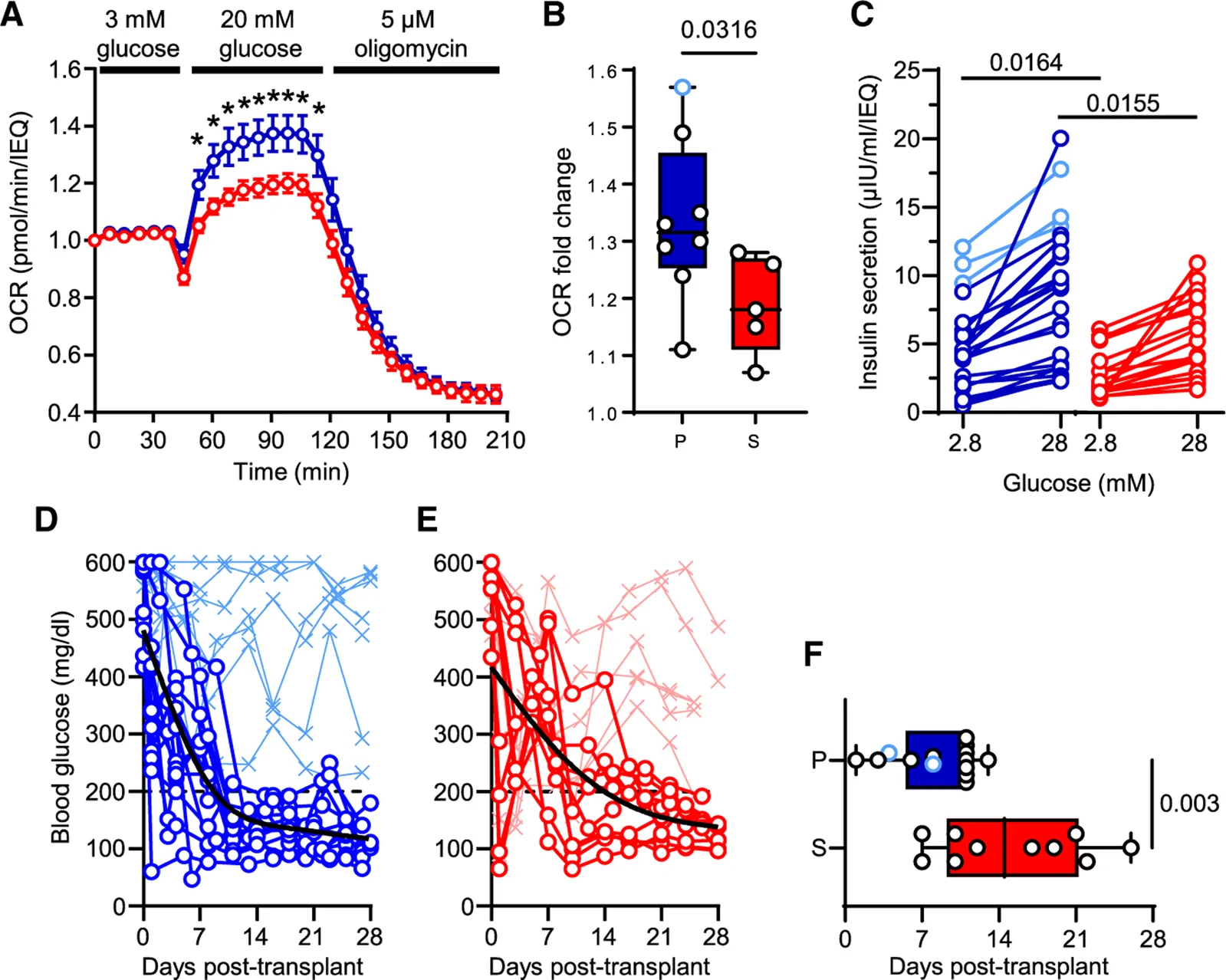
Improved vitality and function of human islets carrying the protective *INS* variant (A) OCR of human donor islets with protective (blue, *n* = 7 of which one is homozygous protective) or susceptible (red, *n* = 5) *INS* variant was determined at basal level, upon glucose stimulation and mitochondrial respiration inhibition. Data are shown as mean ± SEM, *p < 0.05. (B) OCR fold chance of islets with protective (*n*= 8 of which one is homozygous protective) or susceptible (*n*= 5) *INS* variant. (C) Insulin secretion in low and high glucose conditions was compared between islets with protective (*n* = 8 of which one is homozygous protective) or susceptible (*n* = 6) *INS* variant. Each islet preparation was measured in triplicate. (D and E) Blood glucose levels are shown for diabetic mice that were transplanted with human islets carrying either the protective (D; six donors, of which one is homozygous protective, 21 mice) or susceptible (E; four donors, 16 mice) *INS* variants. Mice that did not reverse diabetes (light blue or light red lines with X-symbols) and mice that reversed diabetes (open circles) are shown. The black line shows the mean blood glucose levels of mice that reversed diabetes. (F) Diabetes reversal time is shown for mice transplanted with islets carrying the protective (*n*= 14) and susceptible (*n*= 10) *INS* variant. All data points are shown. For statistical analysis, data from the single and rare homozygous *INS*^*P/P*^ islet donor were combined with those from heterozygous donors, and the homozygous *INS*^*P/P*^ donor is indicated by light blue symbols. Significance was tested by one-tailed t test. See also Table S4.

**Figure 5.**
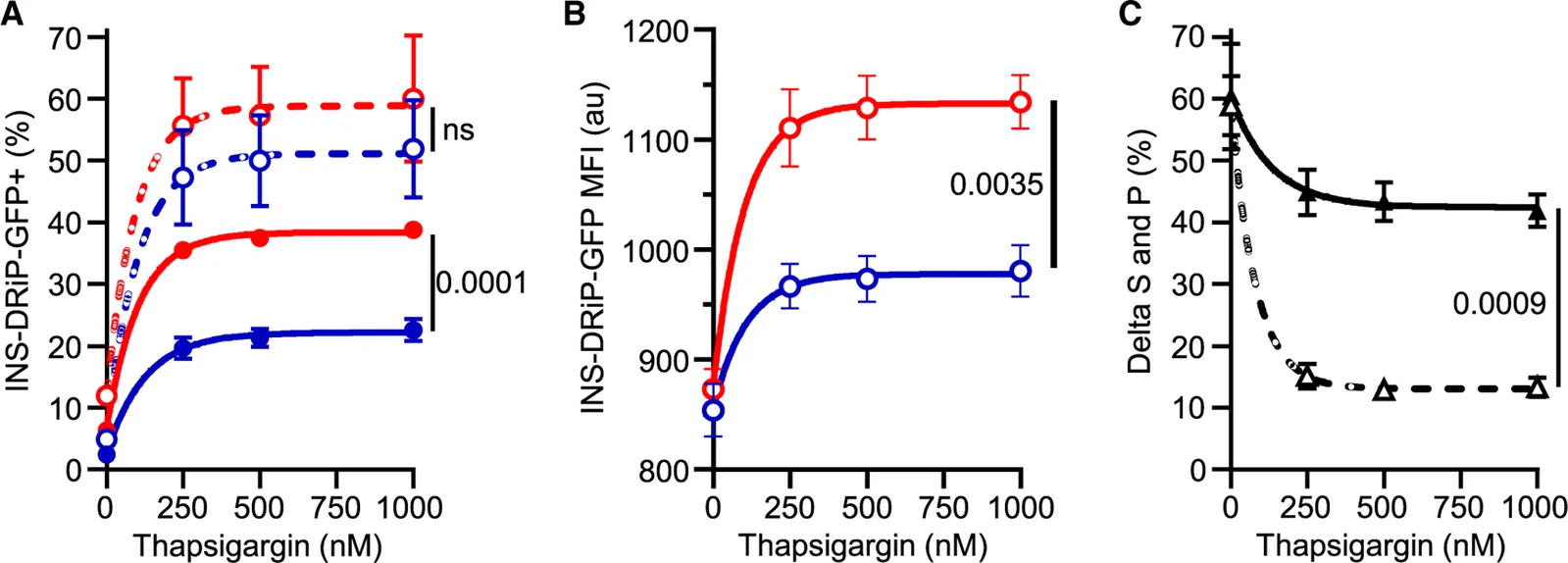
Lower neoantigen expression in surrogate beta cells with protective *INS* variant (A) Percentage of INS-DRiP-GFP+ cell population for the protective (blue) or susceptible (red) *INS* variant, during thapsigargin treatment only (solid lines) and combination treatment with IRE1α inhibitor MKC3946 (dashed lines). (B) MFI of INS-DRiP-GFP+ population for the protective (blue) or susceptible (red) *INS* variant, for indicated thapsigargin concentrations. (C) Difference in INS-DRiP-GFP+ cells between the protective and susceptible *INS* variant for thapsigargin treatment only (solid line) and combination treatment with MKC3946 (dashed line). Data of 3 independent experiments are shown as mean ± SD. Significance was tested by two-way ANOVA. See also Figures S3 and S4.

**Figure 6.**
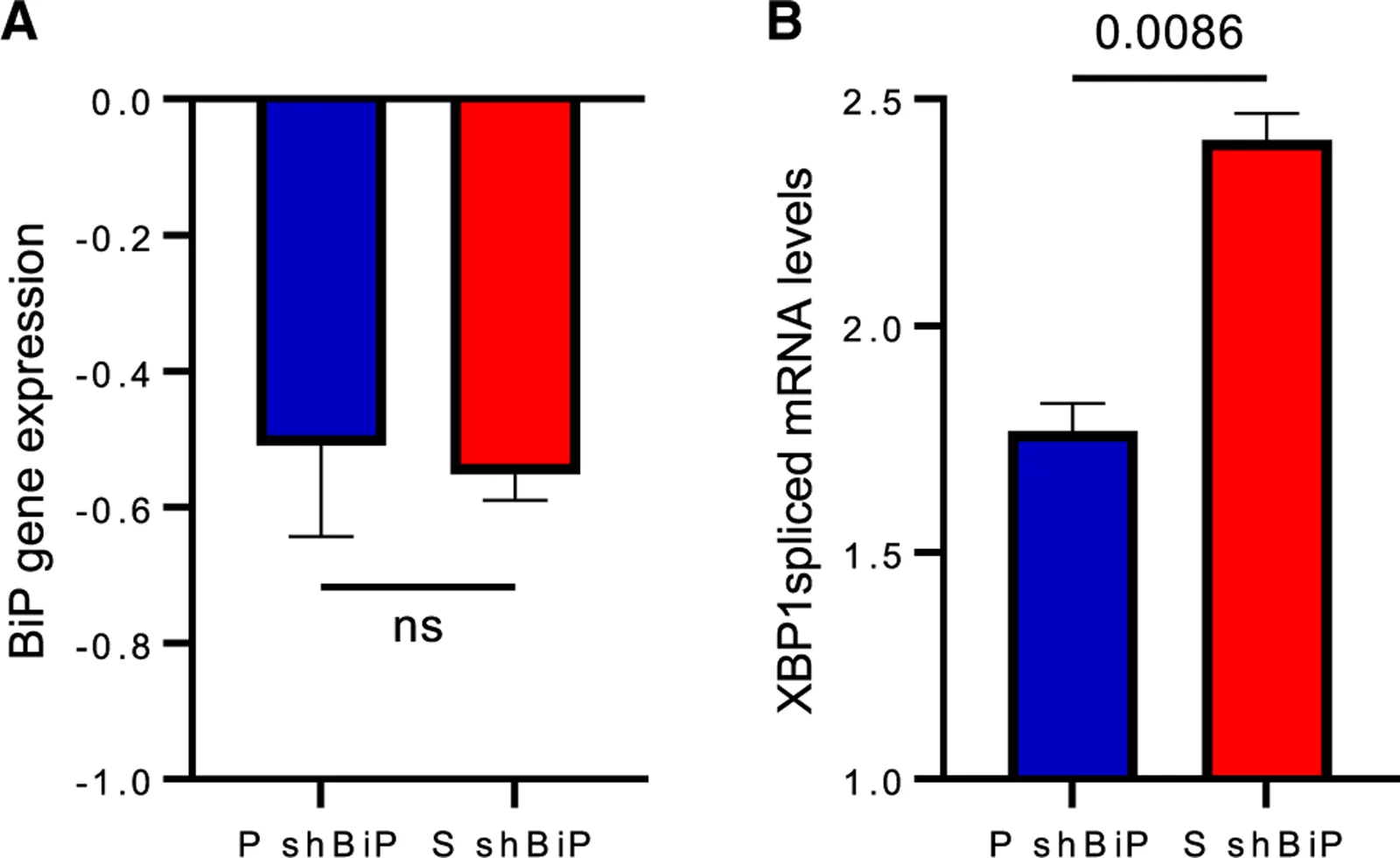
Less ER stress in surrogate beta cells with protective *INS* variant (A) Fold change in relative gene expression levels for BiP. (B) Fold change in relative mRNA levels for XBP1s are shown of surrogate beta cells transduced with *INS*^*P*^ or *INS*^*S*^ and shBiP, as ratio of total XBP1 mRNA. Gene expression and mRNA levels are relative to GAPDH and control transfected for *INS*^*P*^ and *INS*^*S*^ separately. Data of 2 independent experiments are shown as mean ± SD. Significance was tested by two-tailed t test.

**Table:** KEY RESOURCES TABLE

REAGENT or RESOURCE	SOURCE	IDENTIFIER

Antibodies		

rat-anti-C-peptide	DSHB (GN-ID4)	RRID: AB_2255626
donkey-anti-rat-AF647	Jackson Immunoresearch	RRID: AB_2340863

Bacterial and virus strains		

pLV-CMV-INS-DRIP-GFP	This paper	N/A

Biological samples		

Human pancreatic islets	IIDP City of Hope	N/A

Chemicals, peptides, and recombinant proteins		

Thapsigargin	Abcam	Cat#: Ab120286
MKC3946	Cayman Chemical	Cat#: 19152

Critical commercial assays		

Dual luciferase reporter assay	Promega	Cat#: E1910
Insulin ELISA	Mercodia	Cat#: 10–1113-01

Experimental models: Cell lines		

HEK293T	ATCC	Cat#: CRL-3216
K562	ATCC	Cat#: CCL-243

Oligonucleotides		

INS SNP genotyping Fw: 5′-GGAGGGGCTCACAACAGTG Rv: 5′-GTGTCTCCCTGACTGTGTCC	Integrated DNA technologies	N/A
INS taqman assay Fw: 5’-CTGGAGAACTACTGCAACTAGAC Rv: 5’-TGCTGGTTCAAGGGCTTTAT probe: 5’-CTCCTGCACCGAGAGAGATGGA	Applied biosystems	N/A
BiP forward GACGCTGGAACTATTGCTGG BiP reverse CTCCCTCTTATCCAGGCCAT	Sigma-Aldrich	N/A
XBP1us forward GGAGTTAAGACAGCG XBP1us reverse CTGCAGAGGTGCACG	Sigma-Aldrich	N/A
XBP1s forward CTGAGTCCGCAGCAG XBP1s reversed GAGATGTTCTGGAGGGGTGA	Sigma-Aldrich	N/A
GAPDH forward ACAGTCAGCCGCATCTTCTT GAPDH reverse AATGAAGGGGTCATTGATGG	Sigma-Aldrich	N/A

Recombinant DNA		

pSI-Check 2.2-INS 3’ UTR	This paper	N/A

Software and algorithms		

Graphpad Prism 9	Graphpad software	N/A
R version 4.3.1.	R	N/A
SAS Software version 9.4.	SAS Institute	N/A

## Highlights

- A protective *INS* SNP causes accelerated IRE1α-dependent decay of insulin mRNA
- This protective mechanism alleviates ER stress and reduces beta-cell immunogenicity
- Human beta cells with this protective *INS* SNP show better vitality and function
- This discovery puts beta cells at the heart of the disease process leading to T1D
