# Supplementary material for: Genetic protection from type 1 diabetes resulting from accelerated insulin mRNA decay
Source: Cell. Author manuscript; Available in PMC 2026 Oct 22. (PMC13689998; doi:10.1016/j.cell.2025.02.018)
Supplement: Supplementary file 1 [file NIHMS2108285-supplement-1.pdf]

Supplemental figures

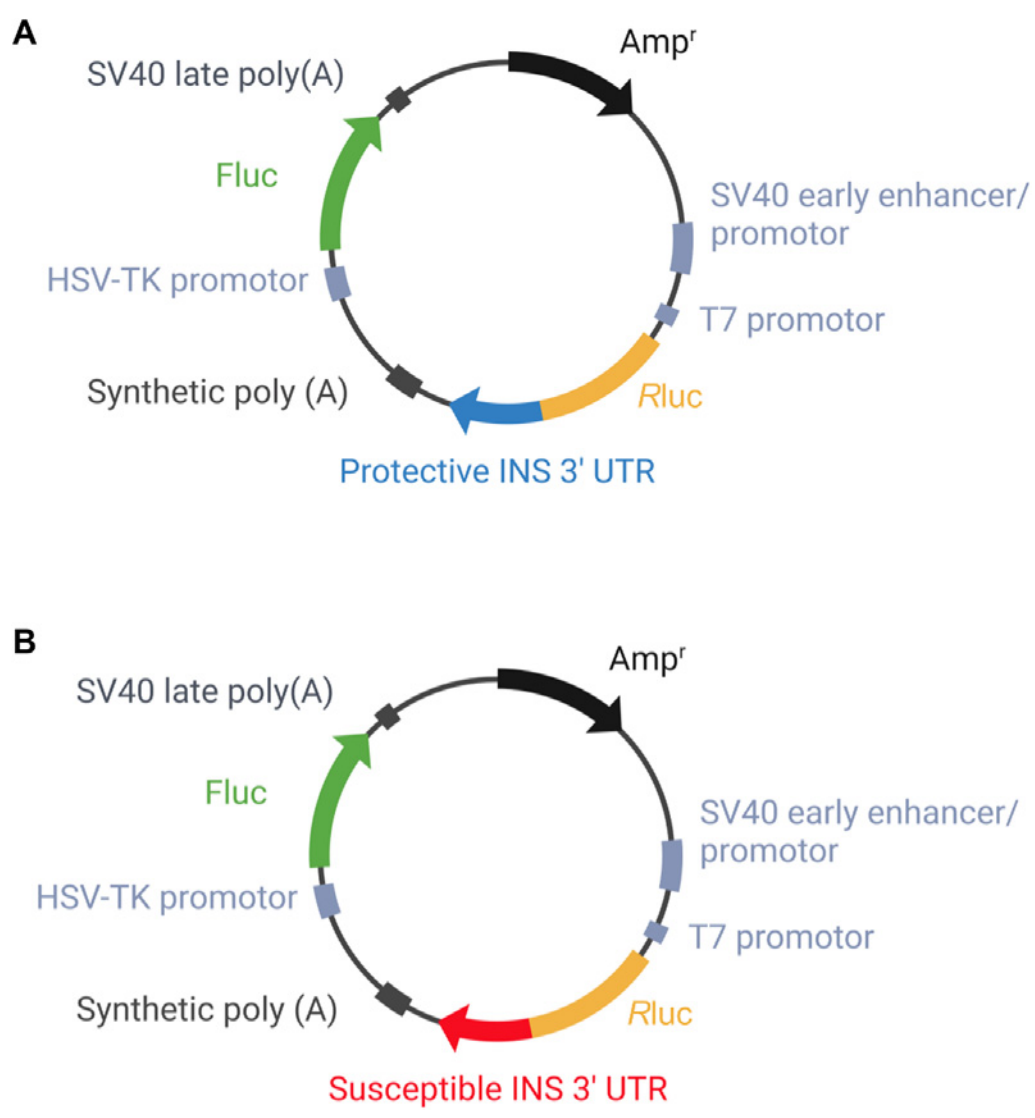

Figure S1. Dual luciferase vector map, related to Figure 2
(A and B) Dual luciferase vector map is shown for *Renilla* luciferase (*R*luc) with protective (A) or susceptible (B) *INS* 3′ UTR. Fluc, Firefly luciferase; AMP^r, ampicillin resistance.

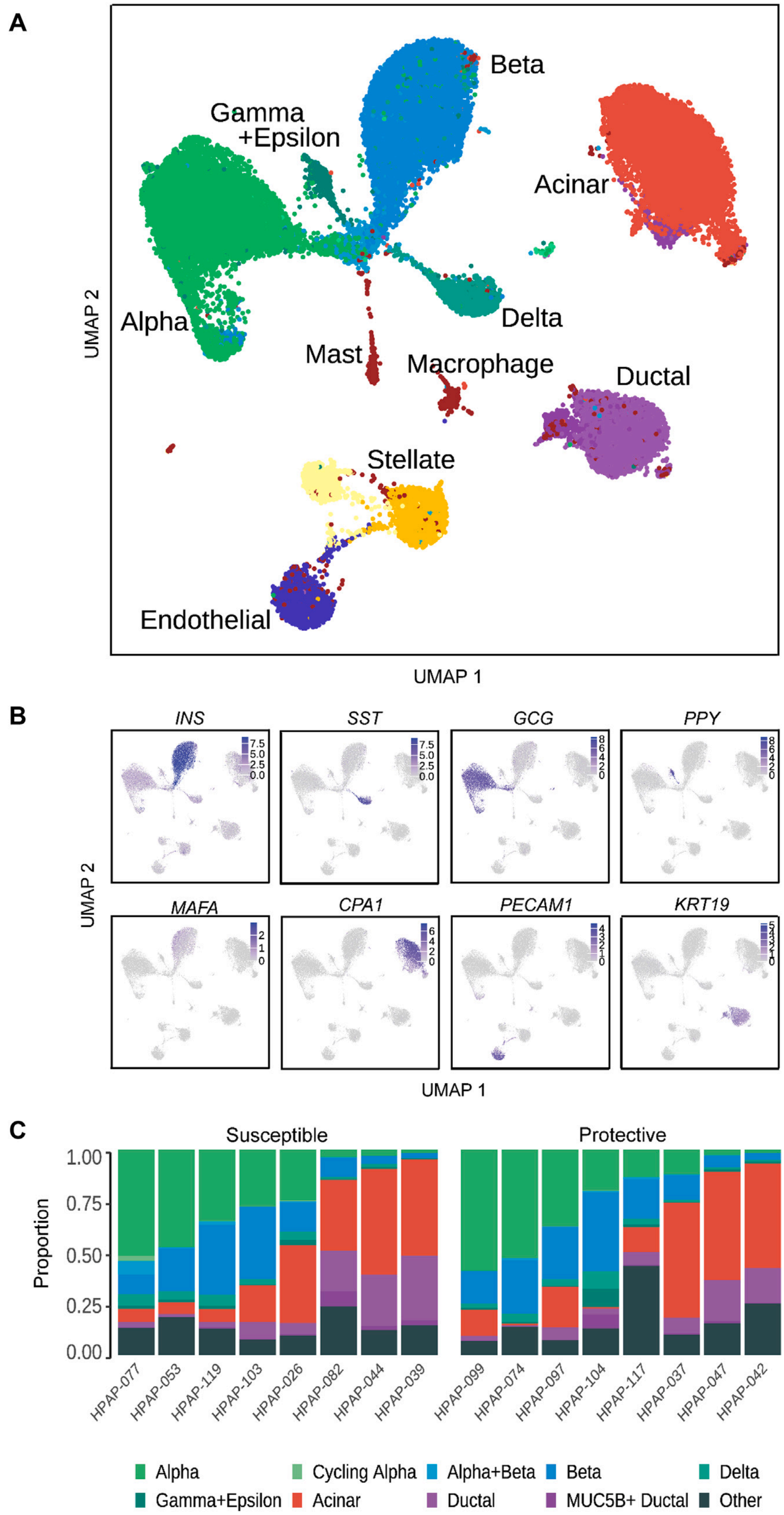

(legend on next page)

Figure S2. UMAP visualization and proportion of cell types in single-cell transcriptomes, related to Figure 3

(A) UMAP visualization of cell types in non-diabetic pancreatic islets. Each point represents a single cell, and colors denote different cell types.

(B) Feature plots showing the expression of key genes across cell types. Each plot represents the expression level of a single gene (*INS*, *SST*, *GCG*, *PPY*, *MAFA*, *CPA1*, *PECAM1*, and *KRT19*) mapped to the UMAP coordinates. Intensity indicates the level of gene expression.

(C) Stacked bar plot showing proportions of cell types for individual donors, separated into those carrying the susceptible (left) and protected (right) *INS* variant. Each bar represents a donor, with colors proportional to cell type counts. Donors are ordered by decreasing proportion of alpha cells within each group. "Other" includes endothelial, mast, stellate, immune, and unassigned cells.

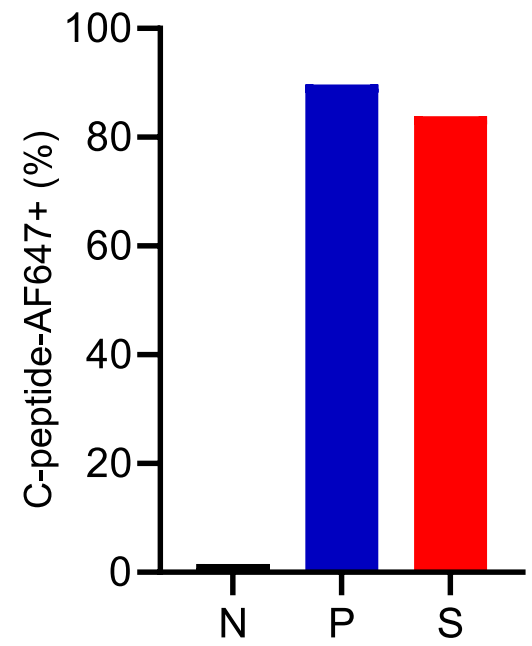

Figure S3. C-peptide expression of surrogate beta cells, related to Figure 5

Percentage C-peptide+ cells are shown for non-transfected (N) cells and cells transfected with protective (P) or susceptible (S) INS-DRiP-GFP vector.

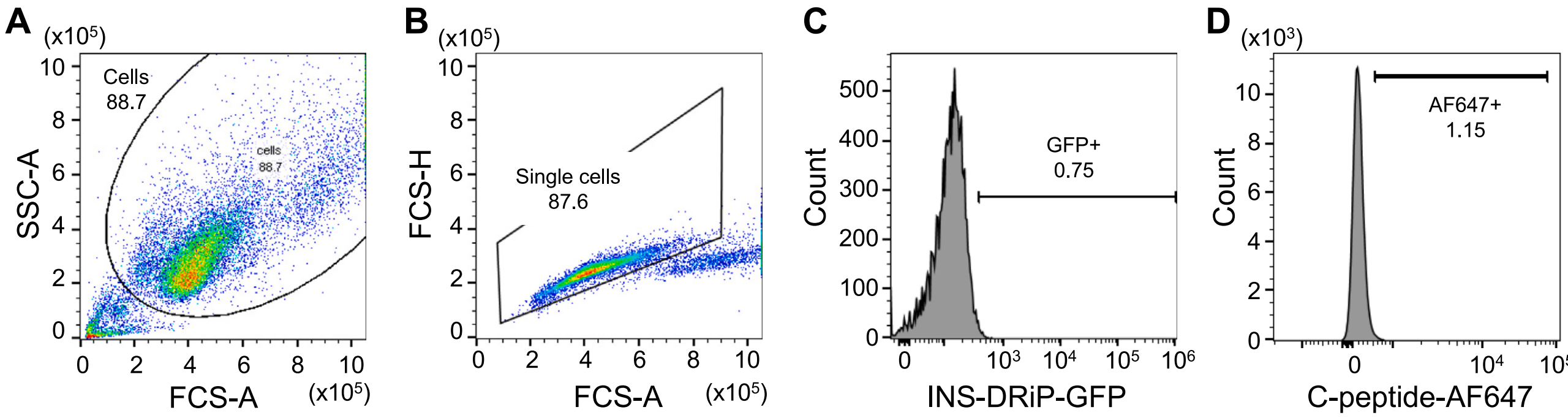

Figure S4. Gating strategy for flow cytometry, related to Figure 5

(A) HEK 293T cells were gated by forward scatter area vs side scatter area (FSC-A/SSC-A).

(B) Single-cell events were selected based on FSC-A/FSC-height (H).

(C) Non-transfected HEK293T cells were used to set the gate for the INS-DRiP-GFP-positive population.

(D) Non-transfected HEK293T cells were stained for C-peptide-AF647 and used to set the gate for the C-peptide-AF647 population.
