# Supplementary material for: Genetic protection from type 1 diabetes resulting from accelerated insulin mRNA decay
Source: Cell. Author manuscript; Available in PMC 2026 Oct 22. (PMC13689998; doi:10.1016/j.cell.2025.02.018)
Supplement: Supplementary file 2 [file NIHMS2108285-supplement-Document_S1__Tables_S1_S4.pdf]

Cell, Volume *188*

Supplemental information

Genetic protection from type 1 diabetes resulting from accelerated insulin mRNA decay

René van Tienhoven, Denis O'Meally, Tristan A. Scott, Kevin V. Morris, John C. Williams, John S. Kaddis, Arnaud Zaldumbide, and Bart O. Roep

Table S1. Raw Ct values of the PCR, related to Figure 1C

time (min)	homozygous susceptible *INS*			heterozygous protective *INS*			homozygous protective *INS*		
0	12.59	12.69	12.55	13.07	13.05	13.14	9.26	9.56	NA
10	12.47	12.43	12.41	13.02	12.97	13.09	11.04	11.29	11.14
20	12.52	12.46	12.60	13.40	13.79	13.77	16.83	16.72	16.73
40	12.96	NA	12.90	13.83	13.38	14.19	12.87	13.08	13.00
80	13.53	13.41	13.89	14.56	14.52	14.53	13.16	13.31	13.39

Table S2. Raw Ct values of the PCR, related to Figure 1D.

INS variant	Ct values without IRE1α			Ct values with IRE1α		
homozygous protective	13.53	13.65	13.62	15.93	15.90	15.98
heterozygous protective	13.72	13.79	13.83	15.69	15.72	15.80
heterozygous protective	14.47	14.43	14.42	16.45	16.49	16.52
heterozygous protective	14.24	14.33	14.35	15.86	15.92	16.02
homozygous susceptible	14.04	14.07	14.18	15.72	15.78	15.91
homozygous susceptible	15.41	15.50	15.57	16.86	16.77	16.89
homozygous susceptible	13.99	14.12	14.13	15.31	15.39	15.43

Table S3. Clinical and laboratory characteristics of organ donors used for single cell RNA sequencing, related to Figure 3.

Name	(%), Mean (± 1 SD) , or Median (min, max)[A]				p-value
	n	Protected	n	Susceptible	
Age at death (years)	8	29.3 (±23.5)	8	32.3 (±21.0)	0.791
Sex					0.843
Female	4	50%	5	62%	
Male	4	50%	3	38%	
Body mass index (kg/m^2)	8	25.1 (±7.2)	8	24.5 (±8.2)	0.887
Genetic ancestry[B]					0.354
African	2	25%	0	0%	
Ad mixed American	2	25%	1	13%	
European	4	50%	7	87%	
Circumstance of death					0.576
Cardiovascular event	1	12%	2	25%	
Cerebrovascular event	5	64%	2	25%	
Drug intoxication	1	12%	1	12%	
Other[C]	1	12%	3	38%	
HbA1c (%)	7	5.5 (±0.5)	8	5.6 (±0.6)	0.664
C-peptide (ng/mL)	8	5.1 (±3.6)	8	5.4 (±4.3)	0.884
Autoantibodies detected[D]					---
0	8	100%	8	100%	
$\geq$ 1	0	0%	0	0%	
Pancreas transport duration (hours)	8	13.7 (±5.5)	8	16.9 (±5.1)	0.239
Pancreas weight (% of body weight)	8	0.07 (±0.04)	8	0.08 (±0.04)	0.636
Islet yield (islet equivalent count)	8	266,970 (±162,051)	8	249,596 (±187,432)	0.846
Islet viability (% viable)	8	94.5 (84.0, 96.0)	8	92.0 (91.0, 95.0)	0.170
Islet stimulation index	8	1.22 (±0.54)	8	1.41 (±0.58)	0.519

[A] % reported for all categorical variables; use of mean or median based on evaluation of normal distribution

[B] as defined by 1000genomes project

[C] Includes drowning (3) and other traumatic injury (1).

[D] All organ donors tested negative for antibodies to glutamic acid decarboxylase, islet cell antigen 2, insulin, and zinc transporter 8.

Table S4. Clinical and laboratory characteristics of organ donors, related to Figure 4

***INS* variant**		**Protective (n=10)**	**Susceptible (n=8)**	**p-value**
Donor sex (%)	Female	20	25	0.999
	Male	80	75	
Donor age (years)		48.9 ± 9.1	54.0 ± 5.0	0.176
Body mass index (kg/m^2)		31.6 ± 6.2	29.0 ± 4.4	0.326
Hemoglobin A1c (%)		5.96 ± 1.3	6.23 ± 0.7	0.611
Islet purity (%)		79 ± 3	80 ± 5	0.700
Cold ischemia time (hours)		9.2 ± 3.4	7.3 ± 2.6	0.219
